# SiMPull-POP: Quantification of Membrane Protein Assembly via Single Molecule Photobleaching

**DOI:** 10.21769/BioProtoc.5560

**Published:** 2026-01-05

**Authors:** Ryan J. Schuck, Alyssa E. Ward, Francisco N. Barrera, Rajan Lamichhane

**Affiliations:** Department of Biochemistry & Cellular and Molecular Biology, University of Tennessee, Knoxville, TN, USA

**Keywords:** Single-molecule, TIRF microscopy, Photobleaching, DIBMA, Oligomerization, Membrane proteins

## Abstract

Traditional methods for studying protein–protein interactions often lack the resolution to quantitatively distinguish distinct oligomeric states, particularly for membrane proteins within their native lipid environments. To address this limitation, we developed SiMPull-POP (single-molecule pull-down polymeric nanodisc photobleaching), a single-molecule technique designed to quantify membrane protein oligomerization with high sensitivity and in a near-native context. The goal of SiMPull-POP is to enable precise, quantitative analysis of membrane protein assembly by preserving native lipid interactions using diisobutylene maleic acid (DIBMA) to form nanodiscs. Unlike ensemble methods such as co-immunoprecipitation or FRET, which average out heterogeneous populations, SiMPull-POP uses photobleaching to resolve monomeric, dimeric, and higher-order oligomeric states at the single-molecule level. We validated SiMPull-POP using several model systems. A truncated, single-pass transmembrane protein (Omp25) appeared primarily monomeric, while a membrane-tethered FKBP protein exhibited ligand-dependent dimerization upon addition of the AP ligand. Applying SiMPull-POP to EphA2, a receptor tyrosine kinase, we found it to be mostly monomeric in the absence of its ligand, Ephrin-A1, and shifting toward higher-order oligomers upon ligand binding. To explore factors influencing ligand-independent assembly, we modulated membrane cholesterol content. Reducing cholesterol induced spontaneous EphA2 oligomerization, indicating that cholesterol suppresses receptor self-association. Overall, SiMPull-POP offers significant advantages over conventional techniques by enabling quantitative, single-molecule resolution of membrane protein complexes in a native-like environment. This approach provides critical insights into how membrane properties and external stimuli regulate protein assembly, supporting broader efforts to understand membrane protein function in both normal and disease states.

Key features

• Precise determination of membrane protein stoichiometry (e.g., monomer, dimer, oligomer) by directly counting photobleaching steps, overcoming the averaging limitations of bulk assays.

• By incorporating membrane proteins into DIBMA lipid particles (DIBMALPs), this preserves native lipid interactions, offering a more physiologically relevant context for studying protein assembly.

• Sensitively detects ligand-induced or membrane property-driven changes in oligomerization, making it a powerful tool for investigating both constitutive and regulated protein interactions.

## Background

**Figure 1.**
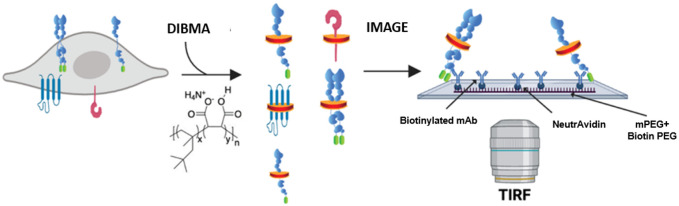
Schematic of single-molecule pull-down polymeric nanodisc photobleaching (SiMPull-POP). Cellular expression of a fluorescently tagged protein of interest, shown here as the GFP-tagged construct, that is extracted via membrane fractionation and solubilized with diisobutylene maleic acid (DIBMA). The solubilized sample is then flowed onto a Biotin-PEG/NeutrAvidin surface, incubated with a biotinylated antibody (“bait”) for the protein of interest (“prey”), and imaged by total internal reflection fluorescence (TIRF) microscopy. Figure modified from Schuck et al. [5].

Experimentally capturing the complexity and dynamic nature of protein–protein or protein–lipid interactions in a native cellular environment remains a challenge [1,2]. Commonly used in situ methods to examine protein–protein interactions (i.e., resonance energy transfer imaging, two-hybrid screens, and co-immunoprecipitation) cannot easily preserve in vivo protein interactions due to the many steps between sample preparation and measurement and are blind to other heterogeneous interactions present in the sample [3]. Moreover, most techniques provide an ensemble measurement of changes in protein assembly rather than a distinct quantification of its oligomeric state. In order to quantitatively explore protein–protein interactions and the influence of lipids on these interactions in a more native context, we optimized and adapted a single-molecule pull-down (SiMPull) protocol previously reported [3,4]. Our advancement of the technique allows for retention of the native membrane environment by incorporating the co-polymer di-isobutylene maleic acid (DIBMA), which spawned the additional name SiMPull-POP (polymeric nanodisc photobleaching). SiMPull-POP is a method by which we can isolate proteins of interest directly from cell lysates and analyze them by total internal reflection fluorescence (TIRF) microscopy at single-molecule resolution (Figure 1).

The sensitivity of TIRF microscopy alleviates the need for large amounts of protein sample, as samples of interest can be visualized in the pM–nM range. In brief, SiMPull-POP begins by capturing diisobutylene maleic acid lipid particles (DIBMALPs) containing protein(s) of interest using a “bait and prey” type scenario constructed in a flow chamber using a microscope slide and a coverslip. In order to create such a capture system, the slide is passivated in a mixture of methoxy polyethylene glycol (mPEG) and biotinylated PEG (biotin-PEG) to prevent nonspecific adsorption of cell lysates and antibodies [6]. The surface is coated with NeutrAvidin, which interacts with biotin-PEG, followed by a biotinylated antibody against the protein of interest. As cell membrane extracts are passed through the flow chamber, the surface-bound antibody captures the protein of interest, while wash steps eliminate any unbound proteins, eliminating the need for laborious purification steps in a time-efficient manner. The sample is then subject to TIRF microscopy, where proteins of interest, bound to the slide surface, are visualized through excitation of a genetically encoded fluorescent protein tag on the protein of interest. By analyzing the fluorescence of individual molecules on the slide, over time, we can count individual photobleaching steps to determine how the protein of interest interacts with other proteins with the same tag. By binning photobleaching behaviors based on the number of steps observed, we are able to obtain a precise quantification of the membrane protein assembly state and begin to characterize factors regulating the assembly process.

## Materials and reagents

**Biological materials**

1. Plasmid: pCMV6-EphA2-GFP (Origene, identifier: RG205725)

2. HEK293T cells (ATCC, CRL-3216)

3. Rabbit anti-EphA2 (D4A2) XP biotinylated (Cell Signaling Technologies, 97535), 20 nM of mAb stock in T50 buffer

4. Goat anti-GFP biotin conjugated (Rockland Immunochemical Inc., 600-106-215), 20 nM of mAb stock in T50 buffer

**Reagents**

1. DMEM (Thermo Fisher Scientific, catalog number: 11965092, 500 mL)

2. FBS (Thermo Fisher Scientific, catalog number: A5256701, 500 mL)

3. PenStrep (Thermo Fisher Scientific, catalog number: 15140122, 100 mL)

4. Opti-MEM reduced serum medium [Thermo Fisher Scientific, catalog number: 31985070 (500 mL) or 31985062 (100 mL)]

5. Lipofectamine 2000 transfection reagent (Thermo Fisher Scientific, catalog number: 11668030); see https://www.thermofisher.com/us/en/home/references/protocols/cell-culture/transfection-protocol/lipofectamine-2000.html for the manufacturer’s recommended reaction volumes and steps

6. Phosphate-buffered saline (PBS) (Thermo Fisher Scientific, catalog number: 10010023, 10× solution diluted to 1×)

7. Protease inhibitors (Fisher Scientific, catalog number: PIA32953)

8. Phosphatase inhibitors (Thermo Fisher Scientific, catalog number: A32957)

9. Diisobutylene maleic acid co-polymer (DIBMA) (Anatrace, catalog number: BMA101, 10% w/v in 1× PBS)

10. Pierce recombinant GFP (rGFP) (Thermo Fisher Scientific, catalog number: 88899, 100 nM stock)

11. NeutrAvidin protein (Thermo Fisher Scientific, catalog number: PI31000, 2 mg/mL prepared stock diluted to 0.2 mg/mL working stock)

12. Protocatechuic acid (PCA)/3,4-dihydroxybenzoic acid (Sigma-Aldrich, catalog number: 37580-25G-F, 100 mM stock in Milli-Q water, pH 9.0)

13. Bacterial protocatechuate 3,4-dioxygenase (rPCO), 300 units (Oriental Yeast Co., catalog number: 46852004)

14. Trolox (Acros Organics, catalog number: 2189400500)

**Solutions**

1. T50 buffer (see Recipes)

2. Lysis buffer (see Recipes)

3. Resuspension buffer (see Recipes)

4. Oxygen scavenging system (OSS) (see Recipes)

5. Trolox solution (see Recipes)

**Recipes**

**1. T50 buffer**

10 mM Tris-HCl

50 mM NaCl

pH = 8.0

**2. Lysis buffer**

50 mM Tris-HCl

250 mM sucrose

250 μM CaCl_2_

1 phosphatase inhibitor tablet (per 10 mL of lysis buffer)

1 protease inhibitor tablet (per 10 mL of lysis buffer)

pH = 7.4

**3. Resuspension buffer**

50 mM Tris-HCl

250 mM NaCl

Glycerol 90/10 (v/v)

pH = 8.0

**4. Oxygen scavenging system (OSS)**

1 mL of Trolox solution

40 μL of PCA

1 μL rPCO

Prepare fresh on the day of the experiment.

**5. Trolox solution**

100 mg of Trolox powder

40 mL of dH_2_O (shake overnight at 4 °C in dark)

Filter with a 0.2 μm syringe filter and store at 4 °C covered with aluminum foil or protected from light. Can be prepared and used for ~2–3 weeks. The solution should be clear; do not use it if coloration occurs.

## Equipment

1. Optima Max-XP ultracentrifuge (Beckman Coulter, catalog number: 393315)

2. TLA55 rotor (Beckman Coulter, catalog number: 366725)

3. Sorvall Legend Micro 21R Centrifuge (Thermo Scientific, catalog number: 75002446)

4. Customized TIRF setup using an Inverted IX73 microscope frame (Olympus, water-immersion)

5. EMCCD camera (Andor Technology)

6. TIRF stage (TIRF Labs)

7. 465 nm cable laser (TIRF Labs)

8. 1″ × 3″ × 1 mm thick quartz slides (G. Finkenbeiner Inc., https://finkenbeiner.com/quartzslides )

9. Rectangular 1–1/2 cover glass (Corning Inc., catalog number: 2980-245)

10. Double-sided tape (Scotch, 3 M)

11. Scalpel (Fisher Scientific, catalog number: 22-444-272)

12. General-purpose grease [Dow Corning, MSC: 31735228 (mscdirect.com)]

13. Kimwipes (11 × 21 cm) [Kimtech Science, catalog number: 34155 (x60)/34120 (x30)]

14. 1.5 mL Eppendorf tubes (Fisher Scientific, catalog number: 05-408-129)

15. Microfuge tube polypropylene 1.5 mL, 9.5 × 38 mm 500 count (for ultracentrifugation) (Beckman Coulter, catalog number: 357448)

16. Air-Tite 1 mL syringes (Fisher Scientific, catalog number: 14-817-119)

17. 25-gauge needles (Fisher Scientific, catalog number: 14-826-49)

18. 27-gauge needles (Fisher Scientific, catalog number: 14-821-13B)

19. Costar 96-well plate, black opaque (Thermo Fisher Scientific, catalog number: 137101)

20. Plate reader (BioTek, model: Cytation 5)

21. Corning CytoSmart Cell Counter (Corning, catalog number: 6749)

22. Evos FLoid cell imaging station (Invitrogen, Thermo Fisher Scientific, catalog number: 4471136)

23. Falcon standard tissue culture dishes, 58.1 cm^2 ^(Fisher Scientific, catalog number: 08-772E)

24. CO_2 _incubator (Panasonic, catalog number: MCO-170A1CUV-PA)

25. 0.2 μm syringe filter (Fisher Scientific, catalog number: 13-1001-06)

## Software and datasets

1. GraphPad Prism, GraphPad Software Inc., https://www.graphpad.com

2. Microsoft Excel, Microsoft, https://www.microsoft.com

3. BioTek Gen5 Software for Imaging & Microscopy, Agilent, https://www.agilent.com/

4. IDL software, NV5 GEOSPATIAL SOFTWARE, https://www.nv5geospatialsoftware.com/Products/IDL

5. IDL Raw-Data-Analysis, Ha-SingleMoleculeLab, https://github.com/Ha-SingleMoleculeLab/

6. Single-molecule software, Ha-SingleMoleculeLab, https://github.com/Ha-SingleMoleculeLab/

7. Calculation to convert steps into % *n*-mer, https://github.com/sgouralis/composition_estimator

MatLab, https://www.mathworks.com/help/install/ug/install-products-with-internet-connection.html

8. Single-molecule photobleaching step binning code, Stefanski et al. [7]. Adapted from the Ha Lab scripts, https://github.com/justmwest/single-molecule-photobleaching/tree/master/count_photobleaching_steps

9. Anaconda Navigator + Spyder software, Anaconda, Anaconda.com

Anaconda 2.6 (older versions can be used through the newest update)

Python 3.12.4 (oldest) to the newest release

IPython 8.25.0 to the newest release

Spyder 5.5.1 to the newest release. Required import packages for Spyder: glob, os, random, numpy as np, sys, matplotlib. pyplot as plt, pandas as pd, from datetime import datetime, from tabulate import tabulate

## Procedure

**A. Plating HEK293T cells and transfection**

1. Seed 500,000 cells/mL in a 10 cm plate with 10 mL of DMEM medium containing 10% FBS and 1% PenStrep.

*Notes:*

*1. It is recommended to plate and transfect at least two 10-cm plates per tested condition.*

*2. Cultured cells can be counted using a system such as the Corning Cell counter used in this protocol [5]. The number of cells seeded may need to be optimized depending on the doubling time. For cultures with short doubling times, seeding 1–2 × 10^6^ cells is recommended.*

2. Allow cells to grow and adhere overnight, stationary, at 37 °C with 5% CO_2 _in an incubator.

3. After overnight incubation, ensure that cells are at 70%–80% confluency.

*Note: If cells are below 70%–80% confluency, allow them to grow longer prior to transfection.*

4. Prepare transfection reactions in 15 mL Falcon tubes in a sterile laminar flow hood, one tube per 10 cm plate.

5. For one plate of cells, the transfection reaction will begin with adding 3 mL of Opti-MEM media to the 15 mL Falcon tube.

6. Add the appropriate volume of plasmid encoding the protein of interest to obtain 15 μg of DNA.

7. Mix by pipetting 20 times and incubate for 5 min.

8. Add 60 μL of Lipofectamine 2000 (4:1 Lipofectamine to μg plasmid DNA ratio) and pipette 30 times.

*Note: Polyethylenimine (PEI) can be used instead of Lipofectamine. For this reagent, a 3:1 PEI (µg) to plasmid DNA (µg) ratio is suggested. See step A10 for more details.*

9. Incubate reaction(s) in a sterile laminar flow hood for 25 min at room temperature.

10. After 25 min, add transfection reaction(s) to 10 cm plate(s) containing cells in a dropwise fashion, gently agitating the solution as the reaction is added.

*Note: The transfection reaction mixture can also be prepared by adding PEI to 500 μL of Opti-MEM, mixing by pipetting 20 times, and incubating for 5 min, followed by the addition of a pre-mixed solution of the plasmid DNA with 500 μL of Opti-MEM. Incubate for 20 min after mixing the two solutions by pipetting 20 times. Then, add dropwise to the 10 cm culture plate while lightly agitating the plate; continue as in step A11.*

11. Allow cells with the transfection reaction to grow overnight in an incubator at 37 °C with 5% CO_2_.

*Note: On the following day, ensure the success of the transfection reaction by visualizing the signal of the fluorescent protein attached to the protein of interest (e.g., EVOS FLoid cell imaging system as used in this procedure). In case there is a low transfection efficiency, the following parameters could be adjusted to help improve the transfection step: (1) Allow cells to grow for an extended time (i.e., an additional 24 h), (2) increase the transfection ratio of Lipofectamine to plasmid DNA to 3:1 or 2:1, (3) increase the amount of plasmid used in the transfection reaction, (4) use a different transfection reagent (PEI as suggested), and/or (5) seed a higher number of cells if the culture is at low confluency for the transfection step. If increasing the transfection reagent ratio, monitor cell viability, as some cell lines may become stressed when exposed to higher levels of these reagents.*

**B. Cell lysis and membrane fractionation**

1. Carefully aspirate media from the transfected cell cultures and gently wash each 10 cm plate with 10 mL of 1× PBS. Repeat twice, aspirating after each wash.

*Note: PBS^++^ (containing 0.1 mM CaCl_2 _and 1 mM MgCl_2_) can be used instead of regular PBS if cells are losing adherence during the wash step.*

**Figure 2.**
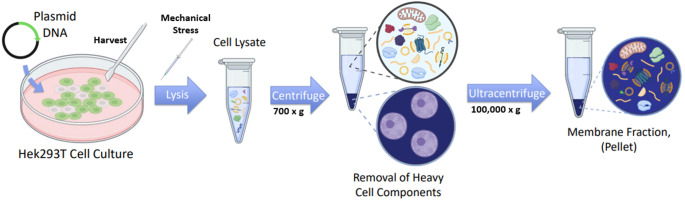
Schematic of cell culture lysis and membrane fractionation. HEK293T cell cultures are seeded into 10 cm culture plates and transfected with plasmid DNA encoding the fluorescently tagged protein of interest. Cells are harvested in lysis buffer by scraping and lysed mechanically by passaging through gauged syringes. The lysate is first centrifuged at low speed to remove large organelles (e.g., nuclei), and the resulting supernatant is then ultracentrifuged to pellet the membrane components.

2. Transfer each 10 cm plate to an insulated benchtop box containing ice.

3. Add 500 μL of lysis buffer (containing fresh protease and phosphatase inhibitors) to each 10 cm plate. Using a sterile cell scraper, collect the cells in the lysis buffer and transfer to a 1.5 mL centrifuge tube. Leave on ice until ready for lysis. An overview of the lysis and membrane fractionation is represented in Figure 2.

*Note: Cultures prepared under the same condition(s) can be collected in a single centrifuge tube; cell harvests from two 10 cm cultures can be combined into a single collection tube, yielding more protein per sample for the solubilization step.*

4. Lyse cells as follows, using 1 mL syringes: pass cells through a sterile 25-gauge needle 20 times, vortex for 30 s, pause, repeat a 30 s vortex, and finally pass cells through a 27-gauge needle 40 times.

5. Centrifuge the cell lysates at 700× *g* to pellet large organelles (i.e., nuclei).

6. Transfer the supernatants from step B5 into 1.5 mL Beckman Coulter ultracentrifuge tubes and keep them on ice until step B7.

*Note: Ultracentrifuge-grade tubes must be used for this step to avoid damage to the rotor and centrifuge due to deterioration of non-ultracentrifuge-grade tubes under high centrifugal forces. To avoid leakage, tubes should be limited to 500–600 μL per tube. Ensure paired tubes for balancing contain an equal amount of volume prior to running. Some samples may need to be split during this step and recombined in steps B10 or B14.*

7. Ultracentrifuge the supernatant at 100,000× *g* (or 40,000 rpm for the TLA-55 rotor) for 1.5 h at 4 °C (minimum 1 h spin time).

8. After the ultracentrifugation, carefully remove the supernatant without disturbing the pellet.

*Note: The pellet obtained here contains the membrane fraction of interest.*

9. Add 400 μL of fresh lysis buffer (containing fresh protease and phosphatase inhibitors) to the pellet.

10. Resuspend pellet by pipetting a few times (recommended, 1 mL pipette tip) and then pass the sample through a 25-gauge needle 10 or more times until pellet is fragmented.

*Note: If applicable, samples originating from identical culture conditions can be recombined here by first resuspending the pellet from one tube and then transferring it to the second tube to resuspend it.*

11. Transfer 400 μL of resuspended pellet(s) to a fresh 1.5 mL Beckman Coulter ultracentrifuge tube.

*Note: This step is important as the initial sample tubes could be compromised from the first spin, pipetting, and exposure to the syringe tip, which could lead to leakage or damage during the following ultracentrifugation.*

12. Ultracentrifuge 400 μL of resuspended pellet samples at 100,000× *g* (or 40,000 rpm for the TLA-55 rotor) for 1.5 h at 4 °C.

13. Carefully remove the supernatant without disturbing the pellet.

*Note: Pellets can be stored at -20 °C overnight or for up to 3–5 days at this step. Allow ~20–50 μL of lysis buffer or enough volume to slightly cover the pellet, parafilm the tube, and store at -20 °C. Resume at step B14 after thawing.*

14. Resuspend pellet in 100–200 μL of resuspension buffer by passing through a 27-gauge syringe at least 10 times until there are no visible large clumps of the membrane pellet remaining.

*Note: The resuspension volume can be varied based on pellet size and adjusted based on the desired concentration. The resuspension buffer can also be changed based on the needs of the experiment (see Troubleshooting section).*

**C. Treatment and solubilization of the membrane pellet with DIBMA**

1. If testing a particular ligand influences the oligomerization of your membrane protein of interest, treat your resuspended pellet with ligand at the desired concentration. Add an equal volume of solvent (matching what the ligand is resuspended in) to samples not containing treatment with ligand (absence of ligand/treatment will serve as the control condition).

Example: As explored in Schuck et al. [5], the protein of interest was the receptor tyrosine kinase, EphA2, that was C-terminally fused to a turboGFP. The oligomerization of this receptor was measured by SiMPull-POP. The membrane fractionation pellet was resuspended as in step B14 with 100–200 μL and then treated for 1 h on ice with 128.4 nM EphrinA-1 Fc prior to the addition of DIBMA. Resuspension of the sample membrane pellet resulted in concentrations ranging between 400 nM and 1 μM.

2. Mix via pipetting 20–30 times. Leave on ice to incubate for 1 h protected from light.

*Note: Samples with fluorescent tags should be protected from light to preserve the photostability of the fluorescent proteins prior to imaging.*

**Figure 3.**
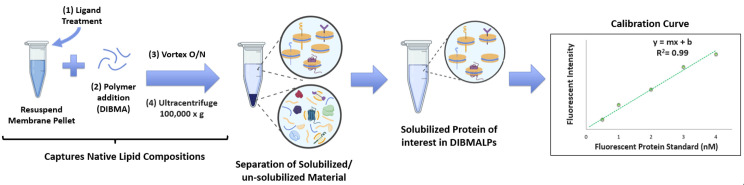
Solubilization and quantification of the protein of interest. The membrane fractionation pellet is resuspended in buffer, treated on ice with ligand, and then incubated overnight with diisobutylene maleic acid (DIBMA). The sample is then ultracentrifuged to remove any unsolubilized materials and quantified by constructing a calibration curve with a representative fluorescent protein standard.

3. After the 1-h incubation, add 1.5 μL of 10% DIBMA (w/v in 1× PBS) per 100 μL of resuspended lysate. Mix by pipetting 20–30 times. An overview of the solubilization steps of the protocol is shown in Figure 3.

*Note: Different membrane proteins may be more or less conducive to solubilization with DIBMA; it is recommended to test other copolymer derivatives to see what is best for your membrane protein of interest. Alternatively, increasing the amount of DIBMA used may result in better solubilization yields for your membrane protein of interest. A final range of 0.015%–2% w/v DIBMA is acceptable. Higher volumes of the polymer in this range can result in more viscous or solidified samples, so testing for the optimal percentage to use is advised.*

4. Parafilm the 1.5 mL centrifuge tubes and place them into a vortex adaptor for 1.5 mL centrifuge tubes. Vortex at medium speed overnight (12–18 h) at 4 °C.

5. After overnight incubation, transfer at least 100 μL of the solution to a fresh 1.5 mL Beckman Coulter ultracentrifuge tube and keep on ice, protected from light, until ready for ultracentrifugation.

*Note: Samples can be prepared in and vortexed overnight in Beckman Coulter ultracentrifuge tubes, then centrifuged the following morning without transferring to new tubes. Ensure exact and equal volumes are pipetted to ultracentrifuge tubes for proper balancing of the ultracentrifuge. It is recommended to weigh each sample using a scale.*

6. Ultracentrifuge samples at 100,000× *g* (or 40,000 rpm for the TLA-55 rotor) for 1.5 h at 4 °C.

7. After ultracentrifugation, remove the supernatant without disturbing the pellet, then transfer the pellet to a fresh 1.5 mL centrifuge tube. Label accordingly.

*Note: The supernatant collected is your final product (DIBMALPs) to work with in the experiment. Keep on ice and in the dark until ready for the next steps.*

**D. Fluorescent calibration curve to measure concentration of DIBMALPs**

1. Prepare fluorescent protein (F.P.) calibration curve (Figure 3) standards using the dilution scheme in Table 1 (stock of fluorescent protein used = 100 nM in 1× PBS).

**Table 1.** Standard solutions for the fluorescent calibration curve

Concentration (nM)	100 nM F.P. stock (μL)	T50 buffer (μL)
0 (blank)	0	110
0.1	0.1	109.9
0.5	0.5	109.5
1	1.1	108.9
2	2.2	107.8
3	3.3	106.7
4	4.4	105.6
5	5.5	104.5
10	11	99
20	20	90

*Note: Duplicates or triplicates of the above solutions should be prepared in order to have repeat measurements on the plate reader. For some samples, a higher standard concentration (e.g., 50 nM) may be needed to improve accuracy.*

2. Mix standards by vortexing and add 100 μL of each solution per well to a Costar 96-well, black opaque plate. Keep protected from the light until plate reader measurements.

3. Prepare a 1:10 dilution of the DIBMALPs collected in step C7 by taking 10 μL of DIBMALP solution and adding 90 μL of T50 buffer.

4. Add 100 μL of diluted samples to the respective wells in the 96-well plate from step D2.

*Note: Duplicates or triplicates of the DIBMALPs solution(s) should be prepared in order to have repeat measurements on the plate reader. The standard curve and samples can also be prepared on a 96-well plate with mixing by pipette and/or shaken prior to reading fluorescence.*

5. Read the fluorescent protein emission of standards and unknown DIBMALPs in accordance with the excitation and emission profile of the fluorescent protein used. Example: If using green fluorescent protein on a plate reader with Gen5 software, select *procedure, read step*, and select the spectrum type *emission*. Excitation is set to *460 nm*, emission start to *495 nm*, emission stop to *600 nm*, and emission step to *1*. Optics position is *top*, read speed is *normal*, and read height is *7.00 mm*. The excitation and emission wavelength variation range should be minimized as much as possible; here, the lowest program setting was 9 nm.

*Note: Depending on the software used, select the gain measurement manually and select the well(s) containing the highest concentration of fluorescent protein stock. In this example, 20 nM of fluorescent protein would be selected.*

6. Read the emission from the samples in the plate. From the collected fluorescent intensities of the samples at 507 nm, subtract the fluorescent intensity (at 507 nm) of the buffer blank from each measured sample.

7. Construct a standard curve graph by plotting fluorescence intensities of the standard curve samples (with blank subtracted from step D6) at 507 nm (y-axis) over known fluorescent protein concentrations (x-axis).

8. Add a linear trend line to the graph and use y = mx + b to solve for the nM concentration of the DIBMALP solution(s) tested. Ensure the final concentration of the solution accounts for the 10× dilution prepared in step D3.

*Note: From the linear regression applied to the graph, check that the R^2^ value is above 95% to ensure confidence in the fit and concentrations extracted from the curve. If the R^2^ value is less than 95%, the calibration range may need to be adjusted (e.g., decrease/increase the range measured) based on the capability of the instrument, or additional points may need to be added to the curve to improve the linearity of the data.*

**E. Flow-cell slide preparation**

1. Clean and passivate microscope slides with mPEG-Silane, including ~2% biotin-PEG-Silane as described [5,6,8,9].

2. Remove passivated slides from the freezer. Warm to room temperature before opening to prevent condensation (~20 min).

3. Using double-sided tape, make two stripes down the long edge of the slide, covering approximately one-third of the PEGylated surface, leaving an empty canal down the middle.

*Note: Do not touch the center of the slide/channel, as this is where the sample will be flowed.*

**Figure 4.**
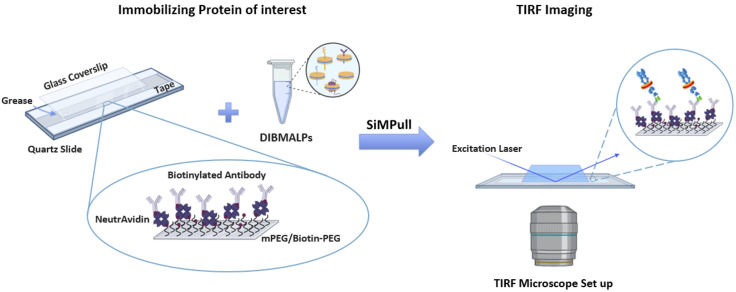
Assembly of the total internal reflection fluorescence (TIRF) imaging slide for immobilizing the protein of interest. Pre-drilled quartz slides are first lined with double-sided tape, after which a thin line of vacuum grease is applied just outside the pre-drilled holes. A glass coverslip is then added on top to seal the flow chamber. The PEGylated surface (mPEG + Biotin-PEG) is treated with NeutrAvidin, a biotinylated antibody specific to the protein of interest, followed by the diisobutylene maleic acid lipid particle (DIBMALP) sample containing the target protein. The prepared flow chamber is subsequently imaged using TIRF microscopy.

4. Add grease via a pre-loaded 1 mL syringe with a modified tip (200 μL pipette tip) in two lines to the slide, just above and outside of the drilled holes.

5. Add a pre-cleaned and passivated coverslip to the top of the quartz slide, ensuring the coverslip is aligned to encompass the two lines of grease and is flush with the long sides of the slide. Adhere the coverslip to the slide by carefully rubbing the blunt end of a scalpel over the coverslip area that is overlaid with the taped area (along the one-third taped portions of the long-edge sides of the slide). Take care to avoid harsh pressure that could cause the coverslip to crack. An overview of the final slide assembly is shown in Figure 4.

6. Cut off excess tape with a scalpel.

**F. SiMPull-POP**

1. Add 70 μL of T50 buffer to the flow cell, ensure there are no leaks, and use a Kimwipe to clean the discharged buffer at the other end of the flow cell.

*Notes:*

*1. In the following steps, after each solution addition, a Kimwipe should be used to collect the discharged solution.*

*2. As a control/precaution, image the flow cell using TIRF microscopy after the addition of buffer to confirm that background spots are either absent or minimal.*

2. Add 70 μL of 0.2 mg/mL NeutrAvidin to the flow cell. Representation of the immobilization surface of the slide is shown in Figure 4.

3. Incubate the slide at room temperature, protected from light, for 10 min.

*Note: For all incubations, ensure that the slide is oriented so that the coverslip is on top and the slide is on the bottom.*

4. Wash out the streptavidin solution in the flow cell by adding 70 μL of T50 buffer. Repeat twice.

*Note: As a control/precaution, image the flow cell using TIRF microscopy after the addition of NeutrAvidin and subsequent washes to confirm that no background spots appear.*

5. Add 70 μL of biotinylated antibody (20 nM in T50 buffer) targeting your protein of interest to the flow cell.

6. Incubate the slide at room temperature, protected from light, for 20 min.

7. Wash out the antibody solution in the flow cell by adding 70 μL of T50 buffer. Repeat a total of two times.

*Note: As a control/precaution, image the flow cell using TIRF microscopy after the antibody incubation and subsequent wash steps are complete. Ensure the number of molecules detected is minimal.*

8. Add 70 μL of the DIBMALPs sample to the flow cell (using the concentration of the fluorescent protein of interest from step D8; dilute to a concentration between 100 pM and 10 nM in T50 buffer).

*Notes:*

*1. The sample concentration should result in the detection of ~200–600 selected molecules per field of view to ensure sample immobilization is greater than the background/nonspecific molecule detection and to avoid oversaturating the slide, causing pixels to overlap, which can convolute data analysis.*

*2. Constructs containing brighter versions of fluorescent proteins (e.g., eGFP) typically work best at a concentration of 100–200 pM. Alternatively, constructs containing less bright fluorescent proteins (e.g., turboGFP) may require concentrations between 1 and 10 nM. Samples should be first tested at a lower concentration range and adjusted based on the number of molecules observed via TIRF microscopy. If needed, slides can be re-incubated with a higher sample concentration. Avoid overcrowding the slide with the sample. Individual molecules should be able to be detected without multiple overlapping at the pixel range, and intensities should not cause saturation when excited.*

9. Incubate the slide with the sample at room temperature, protected from light, for 30 min.

10. Wash out the DIBMALP sample in the flow cell by adding 70 μL of T50 buffer. Repeat a total of 3 times.

*Note: As a control/precaution with a fresh/new slide, image the flow cell using TIRF microscopy after the addition of buffer, NeutrAvidin, sample (* ***excluding the biotinylated antibody*** *), and OSS. Wash the sample off the slide a total of 3 times in accordance with step F10 and ensure that no background fluorescence appears during this step.*

11. Add 70 μL of fresh OSS solution. Ensure the sample is shielded from light, then transfer it to the TIRF microscope for imaging. Place the prepared slide onto the microscope stage and attach the prism. Excite the sample with the respective laser for imaging. For details on slide imaging and calibration of the TIRF microscope, see [6,10].

*Note: Prior to sample imaging, perform TIRF microscope calibration with the IDL script and pre-assembled fluorescent bead slide with the excitation laser (in this case, a 465 nm cable laser) for accurate single-molecule identification with the IDL script.*

**Figure 5.**
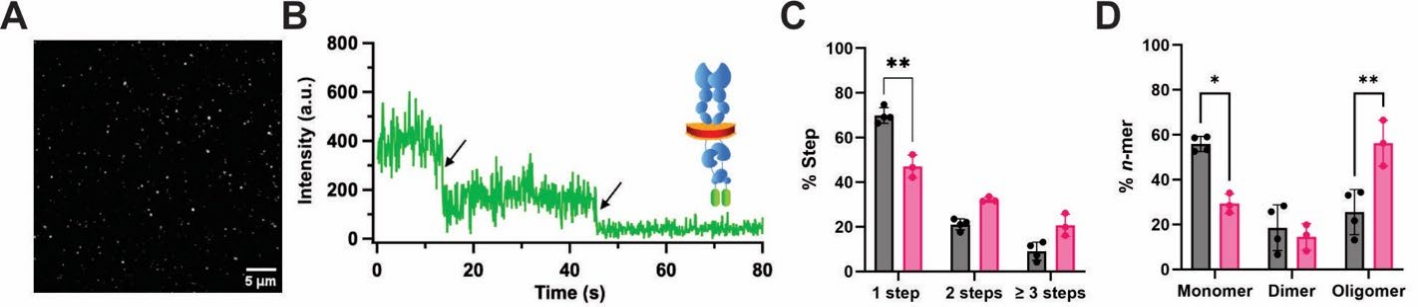
Schematic of single-molecule pull-down polymeric nanodisc photobleaching (SiMPull-POP). (A) Example total internal reflection fluorescence (TIRF) image of individually immobilized fluorescent proteins of interest (scale bar, 5 μm). (B) Single molecule trace of a fluorescently tagged protein of interest showing two photobleaching events marked with black arrows. (C) Step percentage of traces binned as 1-, 2-, or ≥3 steps. (D) Percent oligomer composition after using the composition editor script to convert the % Step data. A two-way ANOVA followed by Tukey multiple comparisons test was run for statistical analysis. Data are mean ± S.D. from three or more independent biological experiments. Figure is modified from Schuck et al. [5]. *p ≤ 0.05, **p ≤ 0.01.

12. Record and extract 10–12 single-molecule videos (intensities over time, 100 ms exposure time, 500 or 1,000 frames) per condition using a custom IDL script from Dr. Taekjip Ha’s laboratory ( https://github.com/Ha-SingleMoleculeLab/Raw-Data-Analysis ). A representative TIRF image of a SiMPull-POP sample is shown in Figure 5A, and a photobleaching trace example is shown in Figure 5B.

*Note: 10–12 videos per condition is a recommendation based on the density of molecules observed while imaging. If each imaging plane contains at least 300 molecules, 10–12 trace files will be enough data for proper analysis. Fluorescent proteins can vary in the length of time to achieve complete photobleaching. It is recommended that initial videos be recorded for 1,000 frames and adjusted to 500 or >1,000 frames based on photobleaching behavior.*

## Data analysis

**A. Analysis of raw photobleaching data**

1. Open Anaconda Navigator and select the Spyder Software after downloading the custom Python code below: https://github.com/justmwest/single-molecule-photobleaching/tree/master/count_photobleaching_steps .

2. Open the CountStepsBlinded_withsave.py tab, and in the indicated line, add the route path to the data file you want to analyze. Note that you will need to direct the route to the folder containing the extracted data files, but not within that folder. In the command prompt line where you have added the data path, press *Shift* and then *Enter* simultaneously to open and visualize the data. In the top-right panel, you can choose the *plots* tab to see the graphed traces in addition to them appearing in the bottom-right panel. The program will prompt you to start a new analysis folder or revisit a previously time-stamped folder. Choose the option to indicate no to start a new folder or *Y* to receive a list of prior analyses. If opening a prior analysis, select the number according to the time point of interest. The program will begin at the last trace opened for analysis or, if starting a new analysis, it will show the first randomized trace plot. For binning the photobleaching traces, there will be an option per trace to bin it as “1–8.” Here, we used “1–3” to represent 1-, 2-, or ≥3-step photobleaching (where the latter included any traces showing 3 or more identifiable photobleaching steps). Once the number of steps observed in the plot is determined, the trace is assigned the respective number by entering *1, 2*, or *3* from the keypad into the prompt line in the bottom-right panel, followed by *Enter*. A new trace is then shown, and the process is repeated for each trace. Only “acceptable traces” are binned. All other traces are “skipped” by pressing Enter on the keyboard with no number assigned in the prompt line. These traces are assigned to a “Rejected” folder under the respective analysis folder. It is also worth noting that traces are randomized based on movie and molecule number. If an error occurs and the previous trace needs to be revisited to be re-assigned or skipped, press *b* on the keypad in the prompt line, followed by *Enter*.

*Note: Traces are deemed acceptable based on the clarity of the photobleaching trace (if steps can be discerned from it). Traces should not exhibit drift (usually seen as a sloped trace), photo-blinking events, intensity in the acceptor trace (if that is not expected), high intensity (≥600, as it is often a sign of protein aggregates), or a return of intensity after photobleaching.*

3. Analyze at least 1,000 traces/molecules per condition tested, with typically ~200–250 accepted molecules/condition (which may require more than 1,000 traces based on molecule accepted/rejected percentage). After analysis completion or to view the number of molecules binned, press *X* on your keypad in the prompt line, followed by *Enter*. If prompted to confirm exit, press *Y* (for yes) and then *Enter*. The data collected will automatically be saved in a folder with the raw data file name followed by *Analysis*, at the file path provided for the data in the left command panel. As stated earlier, the analysis can be reopened later to continue where you left off.

4. After binning the data traces for photobleaching events, open and select the “Sum_counted.py” tab in Spyder. Enter the path to the new data file created in step A3. The path should include the folder with the analysis timestamp in its name.

5. Run the code by pressing *Shift* and then *Enter* simultaneously.

6. After running the code, it will produce and save a summary of the analyses in the same location where your data was initially stored. An individual and total .csv file will be generated. The total.csv file will contain the overall number and percentage of binned traces as 1-, 2-, and ≥3-step events, as well as the total number/percentage of accepted and rejected molecules (an example of a step percent analysis of a SiMPull-POP sample is shown in Figure 5C). In the analysis folder, there will be subfolders for each analyzed video, with additional subfolders for “1–8” and “Rejected.” For this analysis, any traces binned under “1–3” per movie can be accessed in these subfolders. Any skipped traces will be located in the “Rejected” folder per movie. For example, the first movie folder (hel1) will contain the subfolders “1–8” and “Rejected.” Click on the “1” folder to show all traces binned as 1-step photobleaching events. These traces will be in .csv format, with columns representing the intensities of the acceptor and donor traces, along with the frame number. This can be plotted to show the individual traces as needed with intensity (y-axis) and frame number (x-axis), where the latter can be converted to seconds if needed (Figure 5B).

**B. Converting GFP photobleaching steps into oligomeric distribution (% *n*-mer)**

1. Using the analyses folder generated in step A6, identify the number of 1-step, 2-step, and ≥ 3-step photobleaching traces from the total.csv file per analyzed condition.

2. Open a new Excel document. In the first column, enter the number “1” in the A1 cell and drag down the cell to represent the number of 1-step photobleaching events binned for that analyzed condition. Directly under the last “1,” enter a “2” into the cell and drag down to represent the number of 2-step events binned. Repeat this for the ≥3 binned events by using a “3.” For example, if the analyzed condition had 50 binned molecules as 1-step, 25 as 2-steps, and 5 as 3-steps then in the first column of the Excel, there should be 50 cells with a “1,” followed by 25 cells with a “2,” and 5 cells with a “3,” with a total of 80 columns with a 1–3 value in each cell. Ensure no other values are in any other rows or columns.

3. After the values are entered, save this tab/file with the corresponding sample name and save as both a regular Excel workbook file (.xls) and a tab-delimited text (.txt) file.

*Note: To run the composition editor application, the data must be saved as a tab-delimited text file for the application to recognize the data.*

4. Download MATLAB software (R2022–R2024b versions can be used, with installation of the Statistics and Image Processing Toolboxes) along with the composition estimator code application ( https://github.com/sgouralis/composition_estimator ).

5. Open the MATLAB software, followed by the composition editor under the *app* tab.

6. In the composition editor app window, adjust maturation efficiency to 0.7 (if using GFP-labeled fluorophore or to the value associated with the fluorescent protein in use).

7. Press *load step count data*, select a .txt file with sample data of interest, and press *open*.

8. Set the MCMC batch value to *100,000*.

9. Press *create MCMC chain*.

10. Expand the MCMC chain three times in total.

*Note: This expansion step is for settling/minimizing the statistical variance observed when running the MCMC script.*

11. Record the composition of monomers, dimers, and trimers and convert to percentages out of 100% total (as reported on the right side of the composition editor window).

12. Repeat for all samples of interest by pressing *clear step count data* or *reset* and loading another .txt data set of interest.

13. After collecting data from the composition editor, plot the data in GraphPad using the *grouped plot* option.

*Note: Columns represent conditions tested, and rows represent the population of monomers, dimers, and trimers (or 1-, 2-, and ≥3 steps if plotting raw data), for each condition tested. Examples of plotted %Step and %n-mer data are shown in Figure 5C–D.*

14. Run a two-way ANOVA followed by a multiple comparisons test to compare means within rows.

## Validation of protocol

This protocol has been used and validated in the following research article:

- Schuck et al. [5]. Cholesterol inhibits assembly and oncogenic activation of the EphA2 receptor. *Commun Biol*. (Figure 3 and Supplementary Figures 3, 4, 5, 6, 7, and 9)
- Ward et al. [11]. Cholesterol promotes the formation of dimers and oligomers of the receptor tyrosine kinase ROR1. *J Biol Chem.* (Figures 1, 2B, S3–4, and S9)

## General notes and troubleshooting

**Troubleshooting**

**Problem 1:** High background/nonspecific molecules/DIBMALPs detected in the absence of biotinylated antibody targeting your protein of interest.

**Possible causes:** Certain protein constructs are more/less prone to nonspecifically binding to the microscope slide. Sample viscosity can make it “sticky” and lead to nonspecific interactions in the absence of the antibody. The charge of the samples can also lead to electrostatic interactions with the slide, causing nonspecific binding. The passivation/PEGylation of slides may contain “microbubbles” or areas that are not functionalized, allowing the sample to adhere more easily to the slide.

**Solution:** Increasing the number of wash steps at step F10 (as well as the other wash steps) is recommended. Alternatively, you can increase the NaCl concentration of the T50 buffer beyond 50 mM (up to 300 mM NaCl can be used) to address potential electrostatic interactions with the slide/coverslip. Reducing the copolymer in the sample can also help reduce nonspecific interactions. Some buffers can also have intrinsic fluorescence or contain contaminants that may cause background fluorescence. In this case, testing the buffer only in the slide chamber first can help determine whether this is the case. The buffer may need to be changed or filtered if background fluorescence is observed. Imaging the individual components (buffer, NeutrAvidin, and antibody) can also be a step to determine whether any of these stocks may be contributing to background fluorescence/nonspecific binding. If the PEG powder stocks are over a year old or if the socks were exposed to air or above -10 °C for an extended time (stocks should be stored parafilmed in a container with desiccant at -20 °C), this could degrade the functionality of the stock and lead to poor PEGylation of the slide surface. This could lead to a reduced mPEG brush guard, resulting in increased nonspecific interactions between the sample and the slide or reduced sample immobilization. Lastly, 1%–3% (w/v) of bovine serum albumin can be added to the T50 buffer used during the wash steps during the SiMPull-POP slide preparation procedure to help prevent nonspecific binding that may be due to sample “crashing” on the slide.

**Problem 2**: Low yield/solubility of DIBMALPs containing your protein of interest.

**Possible cause:** Certain protein constructs are more or less able to be solubilized by DIBMA.

**Solution:** Test the solubility of your membrane protein of interest with alternative polymers other than DIBMA. Cube-biotech.com offers a variety of polymers other than DIBMA that may work better for different protein constructs. When choosing a co-polymer for solubilization, you need to account for its chemistry, which influences its ability to interact with lipids and, therefore, its ability to extract different types of proteins. The base co-polymer SMA forms tighter nanodiscs suited for more rigid or highly ordered lipid environments, while DIBMA offers a milder charge profile, increased particle generation size, and compatibility with a wider range of lipids and divalent ions [12–14]. Testing can be performed by completing sections A–C and incorporating the different polymers. The percentage used can also be optimized (0.015%–2% range reported); however, higher levels of the co-polymer can cause some samples to gelatinize and/or cause imaging/nonspecific interactions. They should be tested to determine the percentage that works with the conditions/constructs used in the experiment.

**Problem 3:** Too many molecules/DIBMALPs detected on the slide in the presence of the biotinylated antibody targeting your protein of interest.

**Possible cause**: The concentration of Biotin-PEG, the biotinylated antibody, and the fluorescent protein-labeled sample on the quartz slide is too high and may need to be adjusted individually.

**Solution:** Samples should not be recorded at concentrations that saturate the camera's signal. In this case, a new slide will need to be prepared with a lower concentration of the sample. The biotinylated antibody concentration can also be reduced during the incubation step. If needed, the percentage of Biotin-PEG can also be reduced during the passivation step.

**Problem 4:** Too few molecules were detected on the slide in the presence of the biotinylated antibody targeting your protein of interest during TIRF imaging.

**Solution:** Increase the sample concentration. This can be done on the currently imaged slide or a new slide if desired. Likewise, the concentration of the biotinylated antibody can be increased during its incubation step. Additionally, a “pre-incubation” step with the antibody can be used. In this case, incubate the biotinylated antibody and sample (at desired concentrations) for 30 min in a centrifuge tube, and then incubate on the slide (pre-incubated with NeutrAvidin for 10 min) for 20 min. Wash the slide after the incubation three times (70 µL of buffer), followed by the addition of the OSS and image. Lastly, the percentage of Biotin-PEG during passivation can also be increased but should be kept at a minimum to avoid later oversaturation of the slide.

## References

[1] Bagheri Y., Ali A. A. and You M. (2020). Current Methods for Detecting Cell Membrane Transient Interactions. Front Chem. 8: e603259. 10.3389/fchem.2020 .603259 PMC7750205 33365301

[2] Carpenter E. P., Beis K., Cameron A. D. and Iwata S. (2008). Overcoming the challenges of membrane protein crystallography. Curr Opin Struct Biol. 18(5): 581 586 586. 10.1016/j.sbi .2008.07.001 18674618 PMC2580798

[3] Jain A., Liu R., Ramani B., Arauz E., Ishitsuka Y., Ragunathan K., Park J., Chen J., Xiang Y. K., Ha T., .(2011). Probing cellular protein complexes using single-molecule pull-down. Nature. 473(7348): 484 488 488. 10.1038/nature10016 21614075 PMC3103084

[4] Jain A., Arauz E., Aggarwal V., Ikon N., Chen J. and Ha T. (2014). Stoichiometry and assembly of mTOR complexes revealed by single-molecule pulldown. Proc Natl Acad Sci USA. 111(50): 17833 17838 17838. 10.1073/pnas.1419425111 25453101 PMC4273350

[5] Schuck R. J., Ward A. E., Sahoo A. R., Rybak J. A., Pyron R. J., Trybala T. N., Simmons T. B., Baccile J. A., Sgouralis I., Buck M., .(2025). Cholesterol inhibits assembly and oncogenic activation of the EphA2 receptor. Commun Biol. 8(1): e1038/s42003–025–07786–6. 10.1038/s42003-025-07786-6 PMC11897322 40069393

[6] Lamichhane R., Solem A., Black W. and Rueda D. (2010). Single-molecule FRET of protein–nucleic acid and protein–protein complexes: Surface passivation and immobilization. Methods. 52(2): 192 200 200. 10.1016/j.ymeth .2010.06.010 20554047 PMC3321382

[7] Stefanski K. M., Russell C. M., Westerfield J. M., Lamichhane R. and Barrera F. N. (2021). PIP2 promotes conformation-specific dimerization of the EphA2 membrane region. J Biol Chem. 296: 100149 . 10.1074/jbc.ra120 .016423 33277361 PMC7900517

[8] Thakur N., Wei S., Ray A. P., Lamichhane R. and Eddy M. T. (2022). Production of human A2AAR in lipid nanodiscs for 19F-NMR and single-molecule fluorescence spectroscopy. STAR Protoc. 3(3): 101535 . 10.1016/j.xpro .2022.101535 35839771 PMC9293669

[9] Lamichhane R., Liu J., Pauszek III R. and Millar D. (2017). Fluorophore Labeling, Nanodisc Reconstitution and Single-molecule Observation of a G Protein-coupled Receptor. Bio Protoc. 7(12): e2332. 10.21769/bioprotoc.2332 PMC5697779 29170748

[10] Zhao R. and Rueda D. (2009). RNA folding dynamics by single-molecule fluorescence resonance energy transfer. Methods. 49(2): 112 117 117. 10.1016/j.ymeth .2009.04.017 19409995

[11] Ward A., Baeza-Ballesteros L. J., Schuck R. J., García-Murria M. J., Lamichhane R., Mingarro I. and Barrera F. N. (2025). Cholesterol promotes the formation of dimers and oligomers of the receptor tyrosine kinase ROR1. bioRxiv. e660507. 10.1101/2025.06 .19.660507 PMC12800696 41349767

[12] Gulamhussein A. A., Uddin R., Tighe B. J., Poyner D. R. and Rothnie A. J. (2020). A comparison of SMA(styrene maleic acid) and DIBMA(di-isobutylene maleic acid) for membrane protein purification. BBA– Biomembranes. 1862(7): 183281 . 10.1016/j.bbamem .2020.183281 32209303

[13] Grime R. L., Logan R. T., Nestorow S. A., Sridhar P., Edwards P. C., Tate C. G., Klumperman B., Dafforn T. R., Poyner D. R., Reeves P. J., .(2021). Differences in SMA-like polymer architecture dictate the conformational changes exhibited by the membrane protein rhodopsin encapsulated in lipid nano-particles. Nanoscale. 13(31): 13519 13528 13528. 10.1039/d1nr02419a 34477756 PMC8359648

[14] Sawczyc H., Heit S. and Watts A. (2023). A comparative characterisation of commercially available lipid-polymer nanoparticles formed from model membranes. Eur Biophys J. 52: 39 51 51. 10.1007/s00249-023-01632-5 36786921 PMC10039845

